# An updated meta-analysis on the safety and effectiveness of the Contour Neurovascular system

**DOI:** 10.1177/15910199231226280

**Published:** 2024-01-15

**Authors:** Pemla Jagtiani, Georgios S Sioutas, Juan Vivanco-Suarez, Jan-Karl Burkhardt, Visish M Srinivasan

**Affiliations:** 1 School of Medicine, 12298 SUNY Downstate Health Sciences University, New York, NY, USA; 2 189491 Department of Neurosurgery, Hospital of the University of Pennsylvania, Philadelphia, PA, USA; 3 Department of Neurology, 21782 University of Iowa Hospitals and Clinics, Iowa City, IA, USA

We read with great interest the recently published systematic review and meta-analysis by Ghozy et al. entitled “The safety and effectiveness of the Contour Neurovascular System for the treatment of wide-necked aneurysms: A systematic review and meta-analysis of early experience” in Interventional Neuroradiology.^1^ The study reported the effectiveness of the Contour Neurovascular System for wide-necked intracranial aneurysms based on six studies. They found the overall procedure time was 97.27 min (95% confidence interval (CI): 70.07–124.47), the pooled adequate occlusion rate was 84.21% (95% CI: 75.45–90.25), and the overall functional independence rate was 94.74% (95% CI: 87.97–97.79). Thromboembolic events were the most encountered 8.53% (95% CI: 4.78–14.74), subarachnoid hemorrhage (SAH) rate was 0.78% (95% CI: 0.11–5.29), minor stroke rate was 4.35% (95% CI: 1.41–12.63), headache rate was 5.88% (95% CI: 2.83–11.83), and device displacement 3.88% (95% CI: 1.62–8.97).^1^ To our knowledge, several additional studies^2–4^ on this topic have been published since Ghozy et al.'s study was published online in 2022. Therefore, a meta-analysis with updated data is valuable to further clarify the effectiveness of the Contour Neurovascular System as a treatment option for wide-necked intracranial aneurysms, especially as U.S.-based physicians look to evaluate the device in advance of the publication of its FDA IDE trial (NCT04852783).

We followed similar methods to those in the previous meta-analysis.^1^ Guidelines were outlined in the preferred reporting items for systematic reviews and meta-analyses statement (Supplementary Figure 1). A comprehensive literature search was performed in PubMed, Scopus, Web of Science, and EMBASE to identify any articles published between 1 January 2020 and 12 October 2023. The following search strategy was tailored for each database, (“intracranial aneurysm” OR “intracranial aneurysms” OR “intracerebral aneurysm” OR “intracerebral aneurysms” OR “cerebral aneurysm” OR “cerebral aneurysms” OR “brain aneurysm” OR “brain aneurysms”) AND (“Contour Neurovascular System” OR “Contour” OR “Cerus Endovascular”). Our results were obtained by merging studies from the original meta-analysis with new studies from our recent literature search. We excluded case reports, animal studies, and conference abstracts. Continuous variables were summarized as means ± SDs, whereas categorical variables were summarized as percentages and frequencies. When data were presented as medians, ranges, and interquartile ranges, we estimated the means and SDs by applying previously described methods. We estimated all relative rates based on available data for the variables of interest, the data were handled according to the principles of the Cochrane Handbook. A random effects meta-analysis of proportions was performed with a generalized linear mixed model to calculate pooled rates and 95% confidence intervals (CIs) for each outcome. Pooled estimates for procedure time were also derived from a random effects model with inverse variance weighting. Statistical heterogeneity across studies was assessed with the I^2^ test (>50% implies significant heterogeneity). All the analyses and plots were generated using R statistical software (version 4, 1.3) and R Studio.

The main characteristics of all included studies are summarized in Table 1. A total of 192 patients, with 206 treated aneurysms were included. Among these, 68 patients with 75 aneurysms were included through the incorporation of the three additional studies^2–4^ to the studies included in the previous meta-analysis.^5–10^ Specifically, Dange et al. conducted a multi-center study in India with a single operator, involving 13 patients with 14 aneurysms.^2^ Hecker et al. carried out a single-center study in Australia, emcompassing 34 patients with 40 aneurysms.^3^ Mostafa et al. conducted a single-center study in Germany, comprising 21 aneurysms.^4^ Notably, these studies exhibited a sample size largely comparable to those in the original review.

**Table 1.** Summary of the included studies.

					Aneurysm characteristics						
				Age	Aneurysm height (mm)	Aneurysm width (mm)	Aneurysm neck width (mm)	Dome-to-neck ratio	Parent vessel diameter (mm)	Aneurysm location	
Author, Year	Country	Sample size	Male (%)	Mean SD	Mean SD	Mean SD	Mean SD	Mean SD	Mean SD	Artery	Event
Akhunbay-Fudge, 2019	UK	11	0	65 ± 6.4	7.6 ± 2.7	6.0 ± 2.14	3.7 ± 0.89	2.0 ± 0.4	-	Basilar	4
										MCA	3
										ACOM	1
										SCA	1
										ICA	2
Liebig, 2021	Germany	34	55.9	58 ± 11.4	7.1 ± 3.4	6.3 ± 2.4	4.3 ± 1.4	1.4 ± 0.4	2.6 ± 0.5	AComA	13
										MCA	10
										ICA-PO	1
										ICA-T	1
										BA	8
										IC-PC	1
Wodarg, 2022	Germany	8	37.5	60.1	12.8 ± 7.6	10.3 ± 5.4	5.5 ± 2.5	1.9 ± 1.0	-	BA	4
										MCA	2
										AComA	1
										ICA-PO	1
Bhogal, 2020	UK	3	33.3	67 ± 8.7	7.6 ± 0.62	5.7 ± 2	3.6 ± 0.95	-	-	Anterior circulation	3
Diana, 2022	Spain	15	40	61.2 ± 11.6	6.9 ± 4.1	6.7 ± 2.8	3.9 ± 1.5	-	-	ACoA	7
										BA	2
										ICA	6
Biondi, 2022	France	53 (60 aneurysms)	43	56 ± 8.4	-	5.1 ± 1.4	3.7 ± 1	1.3 ± 0.3	-	Anterior circulation	53
										Posterior circulation	7
Dange, 2022	India	13 (14 aneurysms)	46	52.2 ± 12.2	-	6.14 ± 2.26	5.24 ± 1.19	1.21 ± 0.29	-	MCA	6
										AComA	6
										Basilar	1
										dACA	1
Hecker, 2023	Australia	34 (40 aneurysms)	53	62 ± 3.05	4.8 ± 0.475	4.4 ± 0.45	3.0 ± 0.175	1.3 ± 0.1	-	MCA	15
										Acom/ACA	15
										BA	4
										Pcom	3
										ICA	3
Mostafa, 2023	Germany	21	33	58 ± 13.25	-	-	-	2.05 ± 0.433	-	Anterior circulation	17
										Posterior circulation	4

Our updated meta-analysis demonstrated that the overall procedure time was 76.15 min (95% CI: 43.50–108.81) with significant heterogeneity observed (I^2 ^= 96%, *p* < 0.01) (Figure 1a), probably because the procedure duration definitions were heterogeneous in the newly added studies. The pooled adequate occlusion rate was 0.85 (95% CI: 0.78–0.90) with no heterogeneity observed (I^2 ^= 0%, *p* = 0.64) (Figure 1b), and the overall functional independence rate was 0.96 (95% CI: 0.88–0.99) with no heterogeneity observed (I^2 ^= 0%, *p* = 0.81) (Figure 1c). The thromboembolic events rate was 0.06 (95% CI: 0.04–0.11) with no heterogeneity observed (I^2 ^= 0%, *p* = 0.66) (Figure 2a), the subarachnoid hemorrhage (SAH) rate was 0.01 (95% CI: 0.00–0.04) with no significant heterogeneity observed (I^2 ^= 0%, *p* = 1.00) (Figure 2b), the minor stroke rate was 0.02 (95% CI: 0.00–0.10) with no heterogeneity observed (I^2 ^= 0%, *p* = 1.00) (Figure 2c), the headache rate was 0.01 (95% CI: 0.00–0.22) with no heterogeneity observed (I^2 ^= 0%, *p* = 1.00) (Figure 2d), and the device displacement rate was 0.04 (95% CI: 0.02–0.09) with no heterogeneity observed (I^2 ^= 0%, *p* = 0.83) (Figure 2e).

**Figure 1.**
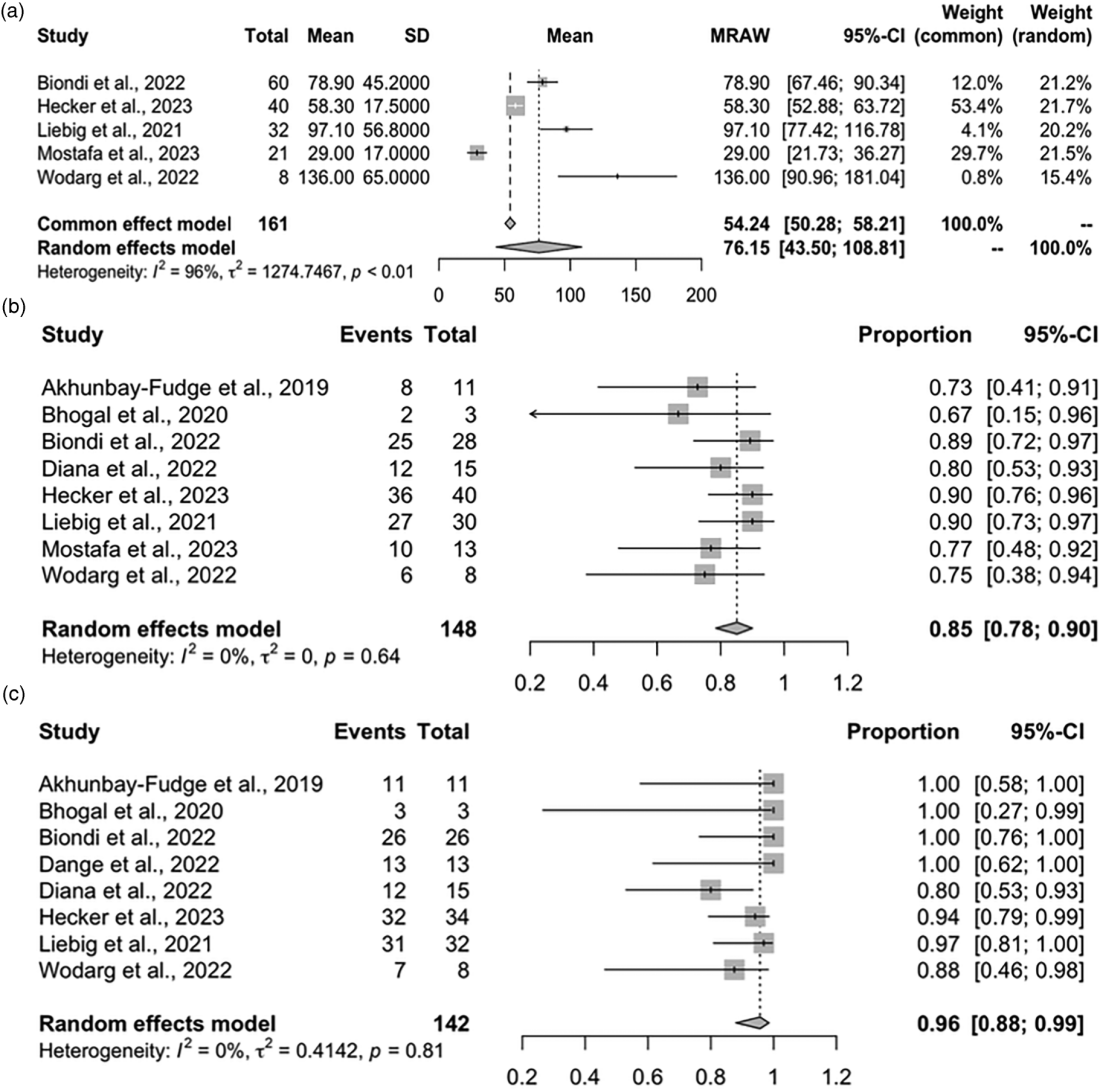
Forest plots for angiographic and clinical outcomes. (a) Procedure time, (b) adequate occlusion (Raymond-Roy), and (c) functional independence (mRS 0–2).

**Figure 2.**
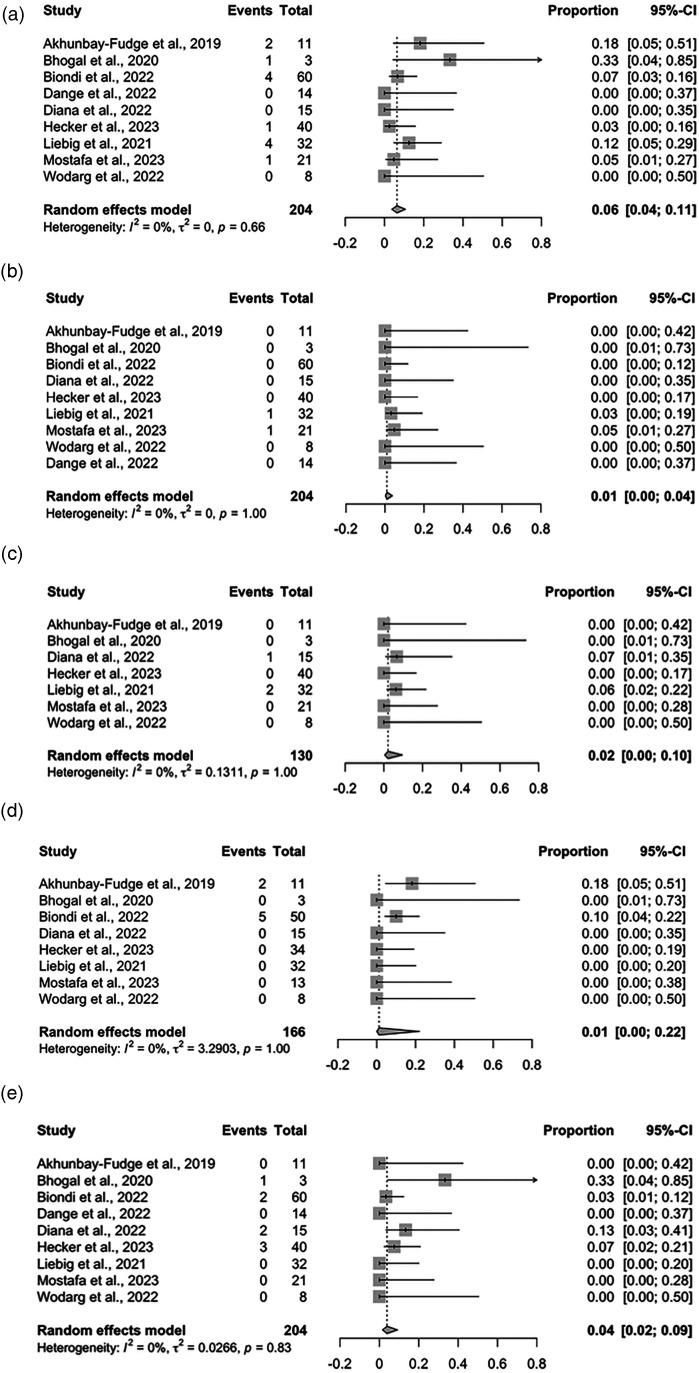
Forest plots for adverse effects. (a) Thromboembolic events, (b) SAH, (c) minor stroke, (d) headache, and (e) device displacement. SAH: subarachnoid hemorrhage.

Woven EndoBridge (WEB) provides an overall occlusion rate of 83.3%, a retreatment rate of 8.4%, and a thromboembolic event rate of 5.6%.^11^ Although extensive data is available for WEB because it is more widely used, data for the Contour Neurovascular System is scarce. Data from our cohort demonstrates a slightly higher occlusion rate of 85% and a slightly higher thromboembolic event rate of 6% for Contour compared to WEB, but these differences were not statistically significant. While the two devices are similar in their makeup of being detachable braided nitinol wires in a single or double layer of mesh, Contour has a few advantages over WEB. The tulip-like structure of Contour makes it easier to deploy the device within the aneurysm sac as it adapts to the bottom of the aneurysm, whereas WEB remains unpredictable in its deployment as it fills out the entirety of the aneurysm sac.^3^

Updated meta-analyses are essential for new devices like Contour, as the data is scarce and continuously evolving. In the absence of clinical trials, it becomes even more essential to continuously moderate findings about the safety and efficacy of a novel device. Based on our current findings, the incorporation of three additional studies resulted in decreased mean procedure time, a slightly higher pooled adequate occlusion rate, and a slightly higher functional independence rate than the original meta-analysis. Moreover, we noted lower thromboembolic events, minor stroke rate, and headache rate, but a slightly higher SAH rate and device displacement rate. We employed a random effects model for all our analyses due to the heterogeneous design, follow-up, and number of participants of the included studies. Most studies defined procedure time as the time frame between the first angiographic run to the last run after deployment but Hecker et al. defined it as the duration of implantation from the first measurement until deployment.^3^ Overall, this suggests a positive trend in the experience with the device with improved outcomes and decreased complications overall.

Several limitations existed in this meta-analysis. There was heterogeneity in follow-up durations and some studies lost patients in their follow-up data. Some outcomes, such as headaches, were not consistently reported. Moreover, most of the enrolled studies were retrospective studies; further meta-analysis with additional prospective studies should be performed to validate our findings.

In conclusion, this updated meta-analysis further demonstrated the safety and effectiveness of the Contour Neurovascular System for treating wide-necked intracranial aneurysms. We anticipate that these updated results will enhance the precision of the data presented by Ghozy et al.^1^

## Supplemental Material

sj-tiff-1-ine-10.1177_15910199231226280 - Supplemental material for An updated meta-analysis on the safety and effectiveness of the Contour Neurovascular system Supplemental material, sj-tiff-1-ine-10.1177_15910199231226280 for An updated meta-analysis on the safety and effectiveness of the Contour Neurovascular system by Pemla Jagtiani, Georgios S Sioutas, Juan Vivanco-Suarez, Jan-Karl Burkhardt and Visish M Srinivasan in Interventional Neuroradiology
