# Supplementary figures and images for: An updated meta-analysis on the safety and effectiveness of the Contour Neurovascular system
Source: Interv Neuroradiol. 2024 Jan 15;32(3):1144–8. doi: 10.1177/15910199231226280 (PMC11571174; doi:10.1177/15910199231226280)

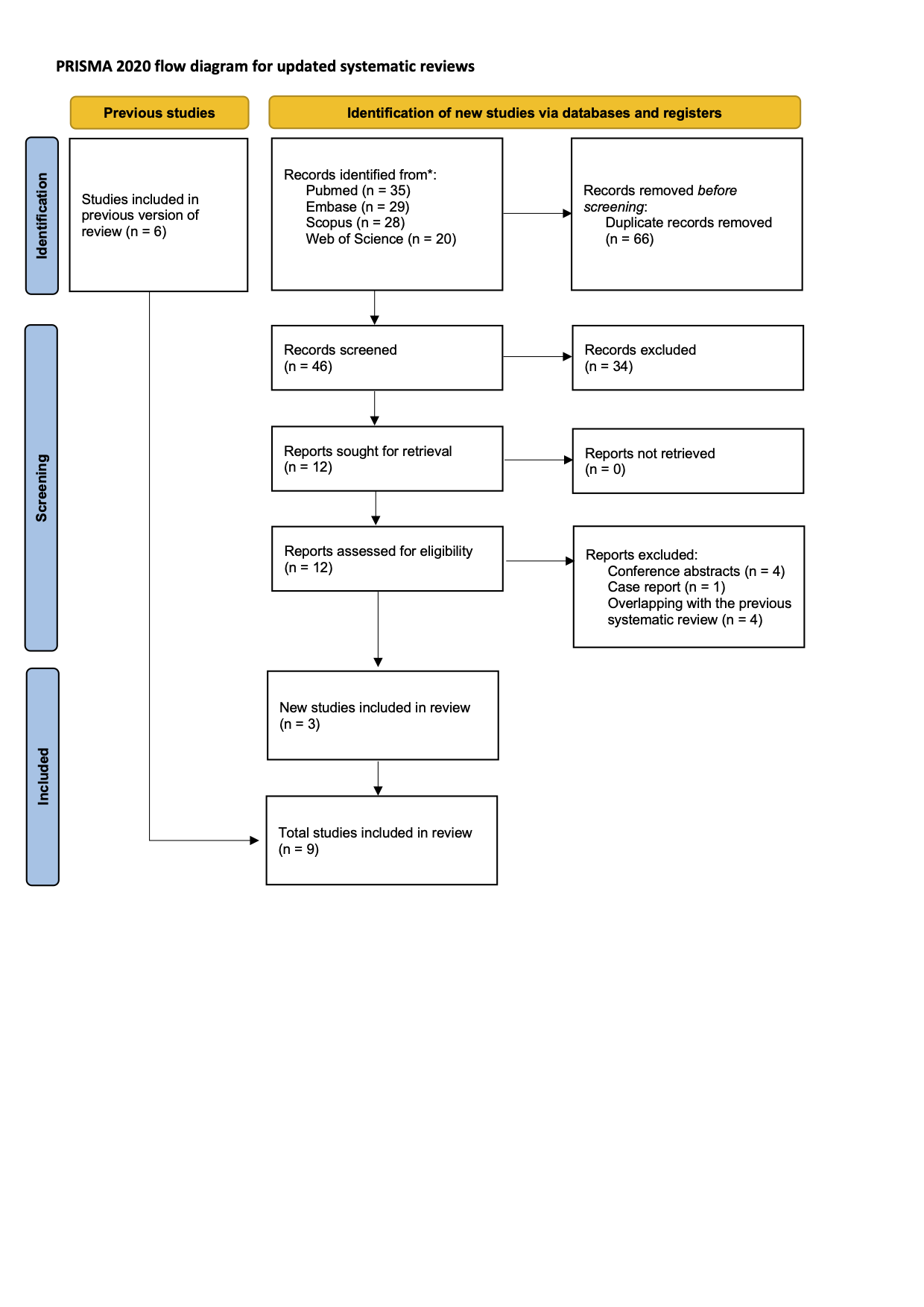

Supplement: sj-tiff-1-ine-10.1177_15910199231226280 - Supplemental material for An updated meta-analysis on the safety and effectiveness of the Contour Neurovascular system [file sj-tiff-1-ine-10.1177_15910199231226280.tiff]
